# Relationship Between First Tarsometatarsal Joint Displacement, Plantar Tarsometatarsal Ligament Morphology, and Hallux Valgus Angle

**DOI:** 10.1002/jfa2.70220

**Published:** 2026-09-25

**Authors:** Mutsuaki Edama, Hiroki Tagawa, Kaito Iwasaki, Kodai Sakamoto, Rinka Goto, Miyu Watanabe, Tomonobu Ishigaki, Makoto Komiya, Hirotake Yokota, Ryo Hirabayashi, Tomoya Takabayashi, Noboru Sato

**Affiliations:** ^1^ Institute for Human Movement and Medical Sciences Niigata University of Health and Welfare Niigata Japan; ^2^ Division of Gross Anatomy and Morphogenesis Niigata University Graduate School of Medical and Dental Sciences Niigata Japan

**Keywords:** first tarsometatarsal joint, hallux valgus, joint displacement, ultrasonography

## Abstract

**Background:**

This study aimed to examine whether the morphology of the articular surface of the first tarsometatarsal (TMT) joint, plantar tarsometatarsal ligament (PTML), and hallux valgus angle (HVA) were associated with plane‐specific TMT joint displacement.

**Methods:**

Nine Thiel‐embalmed cadavers (18 feet) were included. HVA was measured using a goniometer. The capacity for TMT joint motion (mobility) was operationalized as displacement of the first metatarsal relative to the medial cuneiform under manual stress, measured ultrasonographically in the dorsal, plantar, and medial imaging planes. PTML morphology (length, width, and thickness) and TMT joint articular surface morphology (type classification) were assessed, and their associations with plane‐specific TMT joint displacement were analyzed.

**Results:**

No significant differences in dorsal, plantar, or medial displacement were observed between Type III and the other articular surface morphologies after adjustment for multiple comparisons. Similarly, PTML length, width, and thickness were not significantly associated with displacement in any direction. Although HVA was positively associated with plantar displacement before adjustment, this association did not remain significant after FDR correction.

**Conclusions:**

In this small exploratory cadaveric study, no statistically significant associations were identified between first TMT joint displacement and articular surface morphology, PTML morphology, or HVA after accounting for within‐cadaver clustering and multiple comparisons. These findings should be interpreted cautiously because of the small sample size and limited range of HVA severity.

**Level of Evidence:**

Level IV, Descriptive laboratory study

## 1 Introduction

Hallux valgus deformity is characterized by lateral deviation and valgus angulation of the hallux, accompanied by pronation and medial deviation of the first metatarsal [1]. Previous studies have reported a high prevalence of first tarsometatarsal (TMT) joint hypermobility among patients with hallux valgus [2] and an association between TMT joint mobility and hallux valgus deformity severity [3]. These findings suggest that TMT joint mobility may be associated with the presence and severity of hallux valgus; however, its role in the onset or progression of the deformity remains uncertain.

Factors that influence TMT joint mobility include articular surface morphology and ligamentous structures [4, 5]. Regarding TMT joint articular surface morphology, Mason et al. [6] reported three morphological types characterized by one, two, or three articular facets. Specifically, the single‐facet type was exclusively observed in the hallux valgus group, whereas the three‐facet type was found only in the normal group. Furthermore, in our previous study of 100 formalin‐fixed cadaveric feet, the severity of articular cartilage degeneration was significantly lower in the three‐facet type than in other types [4].

The plantar tarsometatarsal ligament (PTML) has been reported as the primary ligament connecting the first metatarsal and medial cuneiform [7], and its sectioning has been demonstrated to induce dorsal displacement of the first metatarsal [8]. Moreover, we previously found that the width and thickness of PTML fiber bundles were negatively associated with articular cartilage degeneration severity in the TMT joint [5]. Nonetheless, the associations of TMT joint articular surface morphology and PTML morphology with the magnitude and direction of TMT joint displacement remain unclear.

Traditionally, formalin‐fixed cadavers allow detailed observation of tissue morphology but have limited joint motion, restricting evaluation of joint and ligament mechanical behavior. The Thiel embalming method preserves life‐like flexibility and permits ultrasonographic imaging, while retaining some mechanical properties similar to those of living tissue [9, 10, 11, 12, 13, 14, 15, 16]. Therefore, Thiel‐embalmed cadavers provide an experimental model for examining associations among articular surface morphology, ligament morphology, and stress‐induced joint displacement using ultrasonography.

Ultrasonography is a noninvasive method for evaluating TMT joint mobility [3, 17]. Specifically, Manceron et al. [3] assessed TMT joint mobility by measuring the separation distance between the first metatarsal and medial cuneiform during passive extension of the metatarsophalangeal joint. Additionally, Maeda et al. [17] evaluated TMT joint mobility by comparing vertical displacement between the first metatarsal and the medial cuneiform during the stance phase of gait. Their findings suggest that ultrasonography is a useful tool for noninvasive quantitative assessment of TMT joint mobility.

Therefore, this study aimed to examine the associations of TMT joint articular surface morphology, PTML morphology, and HVA with plane‐specific TMT joint displacement. We hypothesized that the three‐faceted type of TMT joint would exhibit less displacement than the other types, that greater PTML width and thickness would be associated with less displacement, and that a greater HVA would be associated with greater displacement.

## 2 Methods

### 2.1 Participants

This study included nine Thiel‐embalmed cadavers (18 feet; age, 92 ± 7 years; 12 feet from males and 6 feet from females). The exclusion criteria were as follows: a history of foot or ankle surgery and severe foot deformities.

### 2.2 Assessment of the Hallux Valgus Angle

**FIGURE 1:**
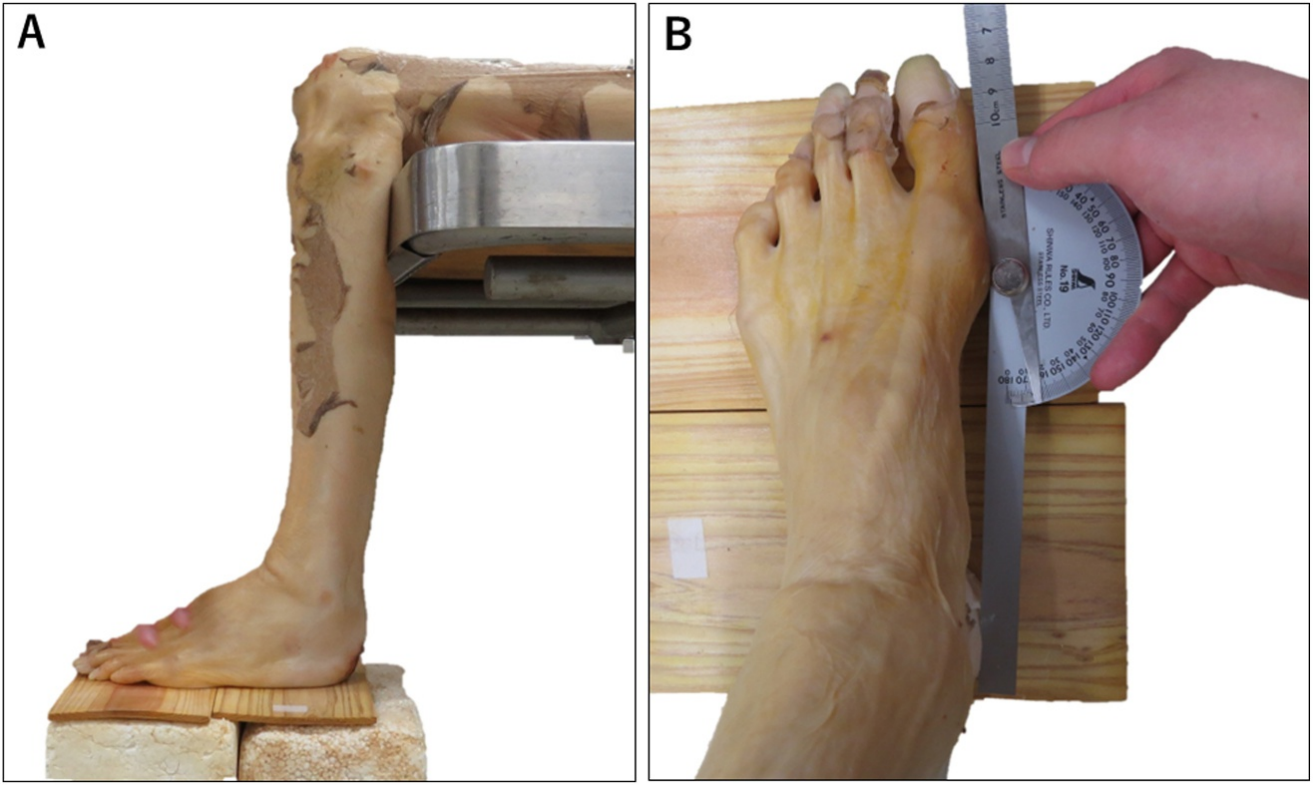
Measurement position and method for the hallux valgus angle. (A) Measurement position: The subject was placed in the supine position, with the lower leg hanging freely off the edge of the table, and the ankle positioned at approximately 0° of dorsiflexion. (B) Measurement of the hallux valgus angle using a goniometer. The stationary arm was aligned with a line connecting the medial border of the calcaneus and the medial eminence of the first metatarsal head, and the moving arm was aligned with a line connecting the medial eminence of the first metatarsal head and the most medial soft tissue of the hallux.

The hallux valgus angle (HVA) was measured using a goniometer according to a previously reported method [18]. The stationary and moving arms were aligned with a line connecting the medial border of the calcaneus and hallux ball and a line linking the hallux ball and the most medial soft tissue of the hallux, respectively. HVA along the medial border of the foot was measured three times, and the mean value was used for analysis. Thiel‐embalmed cadavers were used; therefore, all measurements were performed under non‐weight‐bearing conditions. Measurements were obtained in the supine position with the lower leg hanging freely off the table to standardize limb positioning across specimens (Figure 1).

### 2.3 Assessment of TMT Joint Displacement

The TMT joint exhibits three‐dimensional motion. In this study, TMT joint mobility refers to the capacity for motion and was operationalized as the plane‐specific displacement of the first metatarsal relative to the medial cuneiform under manual stress. Ultrasonography was used to quantify this displacement in the dorsal, plantar, and medial imaging planes; these measurements were not intended to represent complete three‐dimensional joint mobility or instability.

**FIGURE 2:**
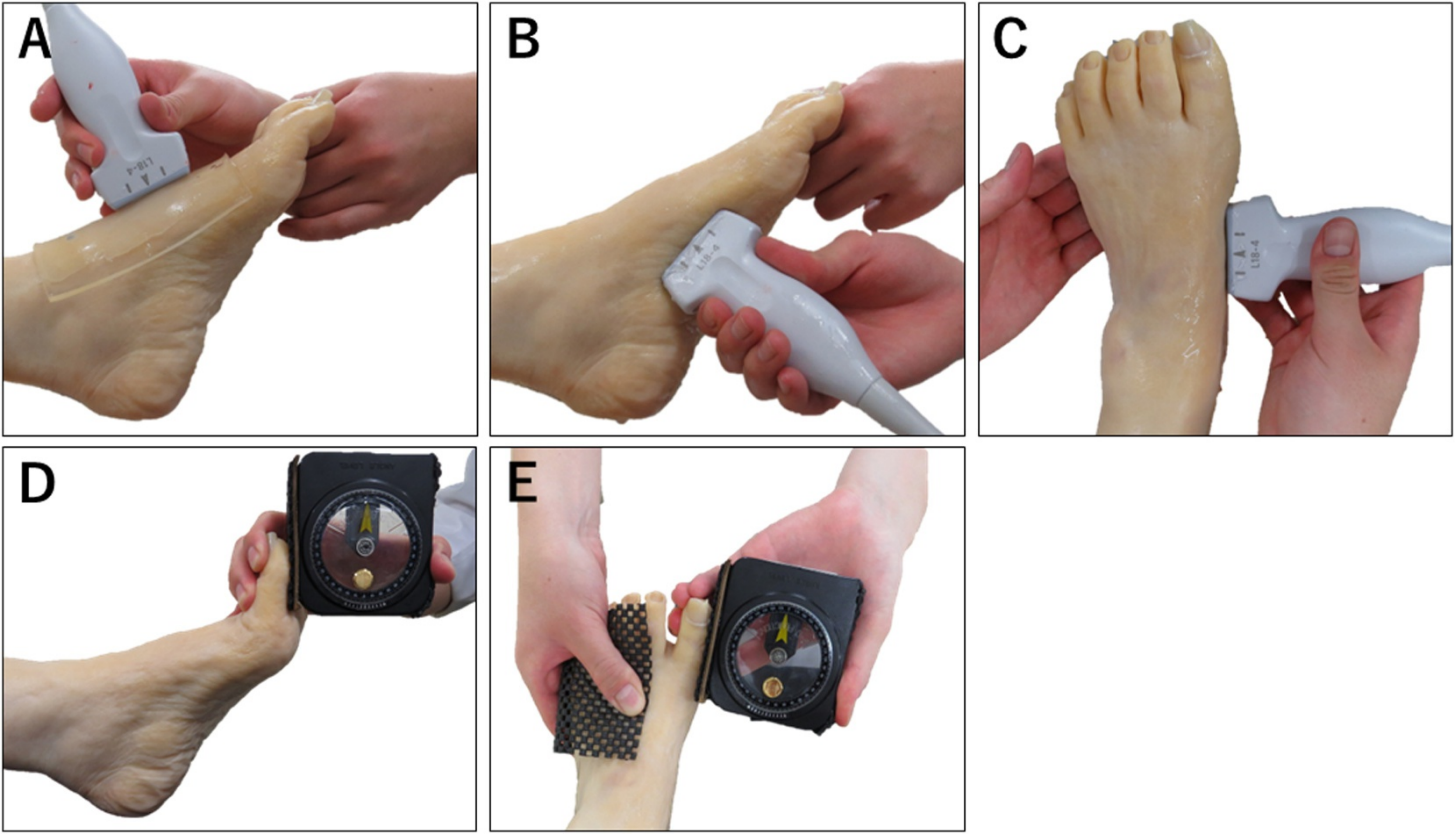
Assessment of first tarsometatarsal (TMT) joint displacement. Ultrasound probe placement: (A) dorsal, (B) plantar, and (C) medial. Stress conditions: (D) maximum hallux extension and (E) maximum hallux adduction.

Plane‐specific TMT joint displacement was evaluated using an ultrasonography system (Sonimage MX1; Konica Minolta, Tokyo, Japan) with a 10‐MHz linear probe. Measurements were performed in the supine position with approximately 40° hip flexion, 40° knee flexion, and 40° ankle plantar flexion. The probe was placed over the TMT joint from the dorsal, plantar, and medial aspects (Figure 2), and the most prominent points of the first metatarsal and medial cuneiform were identified as anatomical landmarks. Manual stress was applied during imaging; maximum hallux extension was applied during dorsal and plantar measurements, and maximum hallux adduction was applied during medial measurements (Figure 2). Maximum hallux extension and adduction were defined as the range of motion achieved until resistance was perceived in each specimen. Two examiners performed each assessment: the same examiner acquired all ultrasonographic images, and the same second examiner applied manual stress to the hallux throughout the study.

**FIGURE 3:**
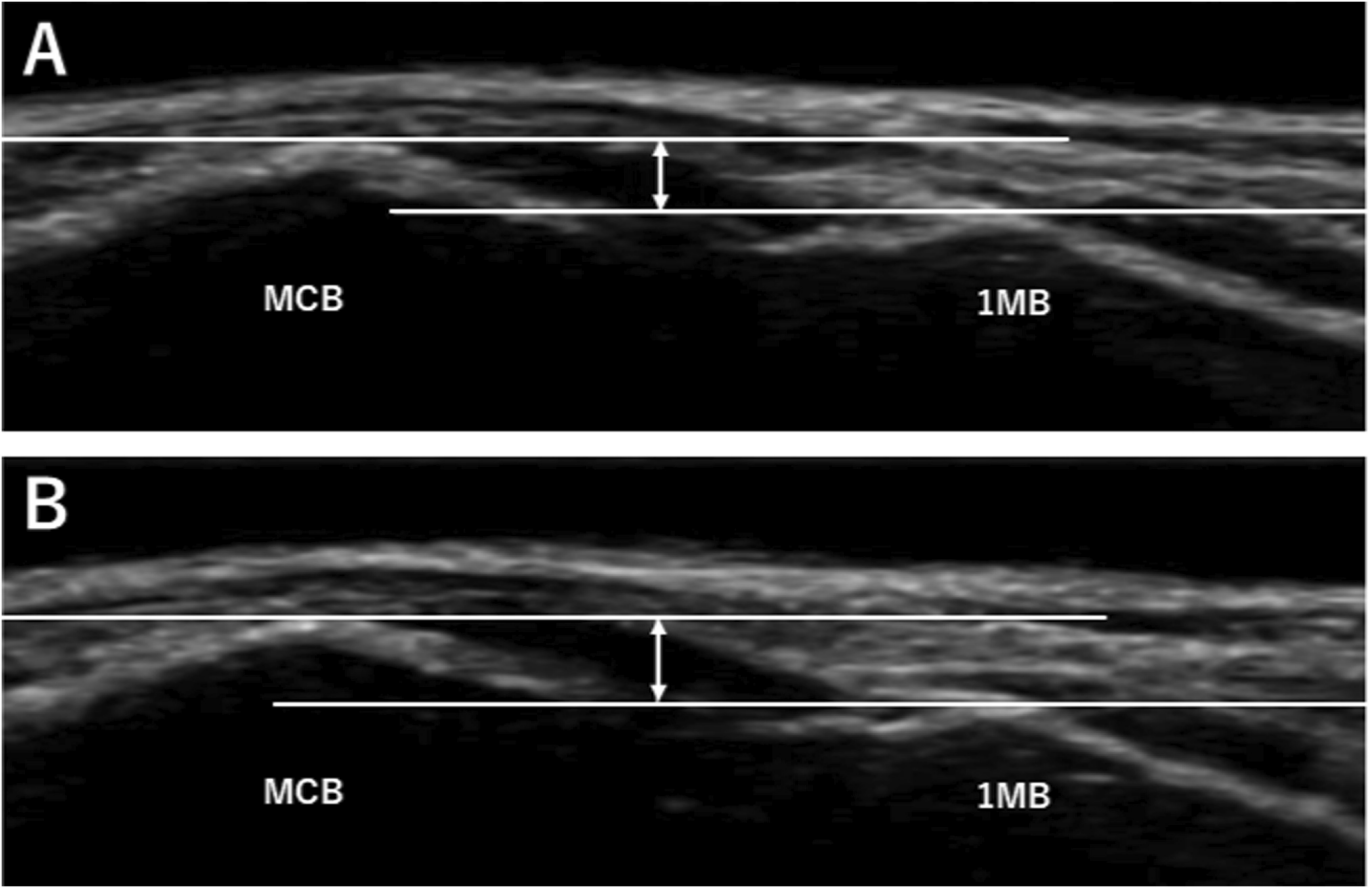
Long‐axis ultrasound images of the dorsal aspect during TMT joint displacement assessment. The white line indicates the horizontal reference line aligned with the most prominent points of the medial cuneiform and first metatarsal. The arrow indicates the vertical distance between the medial cuneiform and the first metatarsal. (A) Before and (B) after stress loading. 1 MB, first metatarsal bone; MCB, medial cuneiform bone.

The acquired images were analyzed using the ImageJ software (National Institutes of Health, Bethesda, MD, USA). A horizontal reference line was drawn at the most prominent points of the medial cuneiform and the first metatarsal, and the vertical distance between the two bones (mm) was measured (Figure 3). An average of three images was used as the representative value. Displacement of the first metatarsal relative to the medial cuneiform was calculated using the following formula:

Displacement(mm)=verticaldistanceunderstress−verticaldistanceatrest.

The order of measurement (dorsal, plantar, and medial) was randomized. To minimize the influence of the ligament's viscoelastic properties, a 2‐min rest period was provided between measurements.

### 2.4 Assessment of PTML Morphology

**FIGURE 4:**
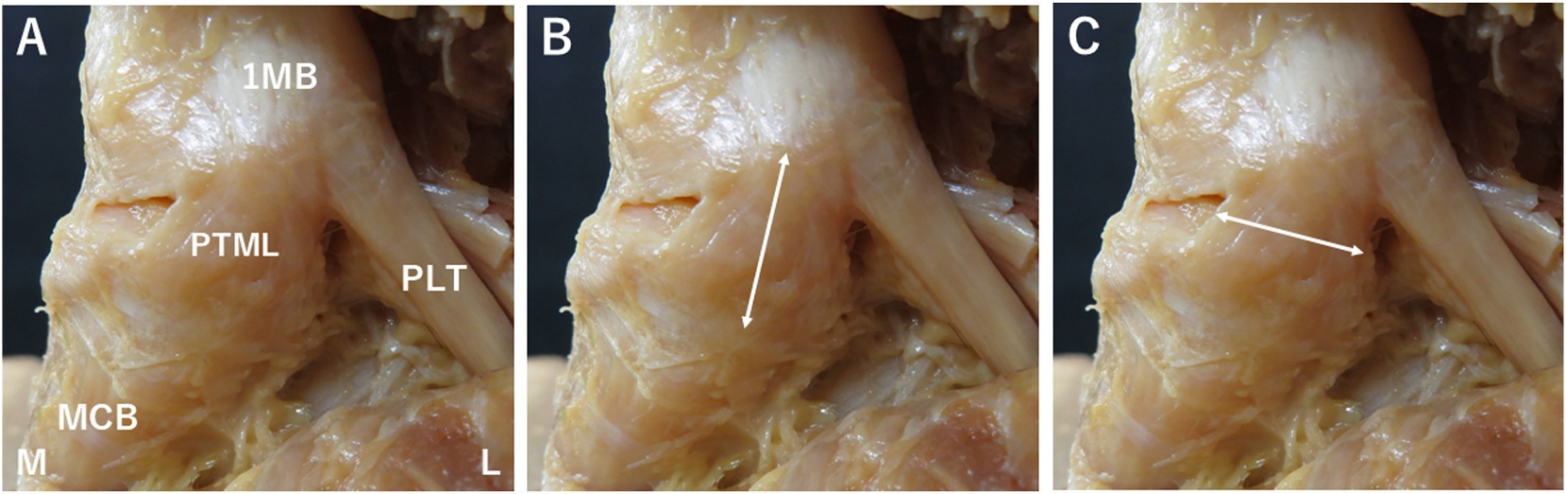
Measurement sites of the morphological features of the plantar tarsometatarsal ligament (PTML). (A) Left foot, plantar view. (B) Measurement site of PTML attachment length. (C) Measurement sites of PTML width and thickness. 1 MB, first metatarsal bone; L, lateral; M, medial; MC, medial cuneiform bone; PLT, peroneus longus tendon; PTML, plantar tarsometatarsal ligament.

PTML morphology was evaluated using digital calipers (model IP54; Shinwa Rules, Niigata, Japan). Based on previous studies [5, 19], the length, width, and thickness of the PTML fiber bundles were measured. The length was assessed at the midpoint of the fiber bundle width, whereas the width and thickness were measured at the distal end of the medial cuneiform. Each parameter was measured three times, and the average value was used for analysis (Figure 4).

### 2.5 Assessment of TMT Joint Articular Surface Morphology

TMT joint articular surface morphology was classified according to a previously reported method [4]. This classification is based on the presence or absence of medial articular surface constriction (“notch”) and a lateral plantar projection protruding inferolaterally from the medial articular surface of the first metatarsal. The articular surfaces were classified as follows: Type I, a single articular surface without a notch or lateral plantar projection; Type II‐a, two articular surfaces with a notch but without a lateral plantar projection; Type II‐b, two articular surfaces without a notch but with a lateral plantar projection; and Type III, three articular surfaces with a notch and lateral plantar projection.

### 2.6 Reliability Analysis

To assess measurement reliability, two measurement sessions were performed for the following assessments: (1) HVA in five cadavers (eight feet; five male and three female feet), (2) TMT joint displacement in four cadavers (eight feet; four male and four female feet), and (3) articular surface morphology and PTML morphology in all nine cadavers (18 feet; 12 male and six female feet). For the repeated TMT joint displacement measurements, the same examiner acquired all ultrasonographic images, while the same second examiner applied manual stress in both sessions. HVA, articular surface morphology, and PTML morphology were assessed by the same examiner in both sessions. The interval between the first and second measurement sessions was at least 1 h.

### 2.7 Statistical Analysis

The reliability of the PTML morphology, hallux valgus angle, and TMT joint displacement measurements was evaluated using intraclass correlation coefficients (ICC [1, 3]). The reliability of the articular surface morphology classification was assessed using Cohen's kappa coefficient. According to the criteria of Landis et al. [20], ICC or kappa values ≥ 0.81 were considered to indicate almost perfect agreement. Data normality was assessed using the Shapiro–Wilk test. The minimal detectable change at 95% confidence (MDC95%) was calculated using the following equation:

$$\text{MDC}=\text{standard}\;\text{error}\times \sqrt{2}\times 1.96$$

The associations of articular surface morphology, PTML morphology, and HVA with TMT joint displacement were examined using linear mixed‐effects models. Cadaver identity was included as a random intercept to account for the non‐independence of bilateral feet from the same cadaver. For the comparison of articular surface morphology, morphology was entered as a fixed effect (Type III vs. other types), and dorsal, plantar, or medial displacement was entered as the outcome in separate models. For the association analyses, PTML length, width, thickness, or HVA was entered separately as a fixed‐effect predictor, and displacement in each direction was entered as the outcome. To account for multiple testing, *p* values were adjusted using the Benjamini–Hochberg false discovery rate procedure separately for the three articular surface morphology analyses, the nine PTML analyses, and the three HVA analyses. Statistical significance was set at an FDR‐adjusted *p* value < 0.05. All statistical analyses were performed using IBM SPSS Statistics version 31 (IBM Corp., Armonk, NY, USA).

## 3 Results

### 3.1 Reliability

For PTML length, width, and thickness measurements, intraclass correlation coefficients (ICCs) exceeded 0.93 for all parameters. The ICC for the hallux valgus angle measurement was 0.95. For ultrasonographic displacement measurements, ICCs were greater than 0.81 in all directions. Regarding the classification of articular surface types of the first metatarsal and medial cuneiform, the kappa coefficient was 1.00. Overall, high reliability was confirmed for all measurements (Table 1).

**TABLE 1:** Intra‐rater, inter‐session reliability and minimal detectable change in the study measurements.

Outcome measures		First	Second	ICC (1,3)	Reliability	MDC 95%
PTML morphology (mm)	Length	14.30 ± 2.44	14.18 ± 2.21	0.98	Almost perfect	1.64
	Width	11.49 ± 1.78	11.11 ± 1.78	0.93	Almost perfect	1.2
	Thickness	0.49 ± 0.11	0.48 ± 0.12	0.95	Almost perfect	0.08
Hallux valgus angle (°)		4 ± 3	5 ± 3	0.95	Almost perfect	3.01
TMT joint displacement (mm)	Dorsal	0.35 ± 0.13	0.36 ± 0.15	0.84	Almost perfect	0.12
	Plantar	0.32 ± 0.22	0.33 ± 0.13	0.83	Almost perfect	0.22
	Medial	0.13 ± 0.08	0.12 ± 0.07	0.81	Almost perfect	0.08

*Note:* Values are presented as mean ± standard deviation.

Abbreviations: ICC, intraclass correlation coefficient; MDC95%, minimal detectable change at the 95% confidence interval; PTML, plantar tarsometatarsal ligament.

### 3.2 Classification of TMT Joint Articular Surface Types

For the first metatarsal, one, nine, three, and five feet were classified as Types I, II‐a, II‐b, and III, respectively. Conversely, for the medial cuneiform, 14 and four feet were classified as Types I and II‐a, respectively, whereas no specimens were categorized as Types II‐b or III. Concordance of the articular surface type between the first metatarsal and medial cuneiform was observed in only one and four feet classified as Types I and II‐a, respectively.

#### 3.2.1 Association Between Articular Surface Morphology and TMT Joint Displacement

Linear mixed‐effects models accounting for within‐cadaver clustering showed no significant differences between Type III and the other articular surface morphologies in dorsal, plantar, or medial displacement after FDR correction (all FDR‐adjusted *P* ≥ 0.751; Table 2).

**TABLE 2:** Comparison of first tarsometatarsal joint displacement between Type III and other articular surface morphologies of the first metatarsal.

Outcome	Type III (*n* = 5) mean ± SD	Other types (*n* = 13)	Estimated difference	95% CI	Unadjusted *p*	FDR‐adjusted *p*
		(Mean ± SD)				
Dorsal	0.32 ± 0.09	0.34 ± 0.14	−0.025	−0.184 to 0.135	0.746	0.751
Plantar	0.72 ± 0.45	0.55 ± 0.27	0.091	−0.189 to 0.371	0.491	0.751
Medial	0.09 ± 0.09	0.08 ± 0.05	0.011	−0.061 to 0.082	0.751	0.751

*Note:* Values are presented as mean ± standard deviation. Estimated differences and 95% confidence intervals were obtained from separate linear mixed‐effects models with cadaver identity included as a random intercept. The estimated difference was calculated as Type III minus other types. *p* values were adjusted across the three outcomes using the Benjamini–Hochberg procedure.

Abbreviations: CI, confidence interval; FDR, false discovery rate.

#### 3.2.2 Association Between PTML Morphology and TMT Joint Displacement

No significant associations were observed between PTML length, width, or thickness and dorsal, plantar, or medial displacement after accounting for within‐cadaver clustering and FDR correction (all FDR‐adjusted *P* = 0.972; Table 3).

**TABLE 3:** Associations of plantar tarsometatarsal ligament morphology and hallux valgus angle with first tarsometatarsal joint displacement.

Predictor	Outcome	B	95% CI	Unadjusted *p*	FDR‐adjusted *p*
PTML length	Dorsal	−0.004	−0.033–0.025	0.779	0.972
	Plantar	0.035	−0.035–0.106	0.305	0.972
	Medial	−0.002	−0.018–0.014	0.770	0.972
PTML width	Dorsal	−0.002	−0.044–0.040	0.920	0.972
	Plantar	0.035	−0.050–0.120	0.388	0.972
	Medial	< 0.001	−0.020–0.021	0.972	0.972
PTML thickness	Dorsal	−0.137	−0.758–0.483	0.645	0.972
	Plantar	0.811	−0.271–1.892	0.127	0.972
	Medial	−0.022	−0.297–0.253	0.866	0.972
HVA	Dorsal	< 0.001	−0.017–0.016	0.976	0.976
	Plantar	0.031	0.0003–0.062	0.048	0.144
	Medial	0.005	−0.002–0.013	0.153	0.229

*Note:* Values are presented as unstandardized regression coefficients (B) with 95% confidence intervals. Separate linear mixed‐effects models included cadaver identity as a random intercept. B represents the change in displacement (mm) per 1‐mm increase in PTML length, width, or thickness and per 1° increase in HVA. *p* values were adjusted using the Benjamini–Hochberg procedure separately for the nine PTML analyses and three HVA analyses.

Abbreviations: CI, confidence interval; FDR, false discovery rate; HVA, hallux valgus angle; PTML, plantar tarsometatarsal ligament.

#### 3.2.3 Association Between HVA and TMT Joint Displacement

The mean HVA was 7.3 ± 4.6° (range, 0.0°–18.0°). Seventeen of the 18 feet had an HVA of < 15°, whereas one foot had an HVA of 18°. HVA was positively associated with plantar displacement before adjustment (*B* = 0.031 mm/°, 95% CI, 0.0003–0.062; unadjusted *P* = 0.048); however, this association did not remain significant after FDR correction (FDR‐adjusted *P* = 0.144). HVA was not significantly associated with dorsal or medial displacement (Table 3).

## 4 Discussion

This study examined the associations of first TMT joint articular surface morphology, PTML morphology, and HVA with first TMT joint displacement while accounting for the non‐independence of bilateral feet and multiple testing. Three main findings were obtained. First, no significant differences in displacement were observed between Type III and the other articular surface morphologies. Second, PTML length, width, and thickness were not significantly associated with displacement in any direction. Third, HVA was not significantly associated with displacement after FDR correction. These findings did not support our original hypotheses.

Regarding the relationship between articular surface morphology and TMT joint displacement, no significant differences in displacement were observed between Type III and the other types after accounting for within‐cadaver clustering and multiple testing. However, this finding should not be interpreted as evidence that articular surface morphology is unrelated to TMT joint displacement because only five feet were classified as Type III and the confidence intervals were wide. The limited concordance of articular surface morphology between the first metatarsal and medial cuneiform may have also influenced the results. In our previous study [4], cartilage degeneration was less severe in congruent Type III joints than in the other congruent types. Similarly, Jung et al. [21] reported that greater articular congruency of the subtalar joint was associated with increased joint stability. These findings suggest that joint congruency, rather than the morphology of the first metatarsal articular surface alone, may be relevant to joint stability. Further studies with larger samples are needed to examine plane‐specific TMT joint displacement according to the combined morphology and congruency of the first metatarsal and medial cuneiform articular surfaces.

Regarding the association between PTML morphology and TMT joint displacement, no significant associations were observed between PTML length, width, or thickness and displacement in any direction after accounting for within‐cadaver clustering and multiple testing. The PTML is the primary ligament connecting the first metatarsal and medial cuneiform [7], and its sectioning has been shown to induce dorsal displacement of the first metatarsal [8]. Furthermore, our previous study demonstrated that greater PTML fiber bundle width and thickness were associated with less severe articular cartilage degeneration in the TMT joint [5]. Based on these findings, we hypothesized that larger PTML dimensions would be associated with reduced TMT joint displacement; however, the present results did not support this hypothesis. Morphological dimensions alone may not adequately represent the mechanical properties of the PTML because ligamentous stabilization may also depend on tissue stiffness, collagen organization, fiber orientation, attachment geometry, and resting tension. Furthermore, the small sample size and wide confidence intervals limit our ability to detect potentially relevant associations. Therefore, the present findings should not be interpreted as evidence that the PTML is not involved in TMT joint stability. Further studies combining morphological assessment with direct measurements of ligament mechanical properties are needed to clarify the contribution of the PTML to TMT joint motion.

Concerning the relationship between HVA and TMT joint displacement, no significant associations were observed after accounting for within‐cadaver clustering and multiple testing. Although HVA was positively associated with plantar displacement in the unadjusted analysis, this association did not remain significant after FDR correction. Manceron et al. [3] reported an association between TMT joint mobility and hallux valgus deformity severity, and a weight‐bearing computed tomography study demonstrated increased TMT joint instability in individuals with hallux valgus [22]. The present study did not confirm these previously reported associations. This discrepancy may be attributable to the small sample size, limited range of HVA severity, non‐weight‐bearing measurement of HVA, and use of elderly cadaveric specimens. Therefore, the observed positive coefficient for plantar displacement should be regarded as exploratory rather than confirmatory. Because 17 of 18 feet had an HVA below 15° C, the present sample cannot adequately characterize associations across the clinical spectrum of hallux valgus severity. Further studies involving larger clinical samples that include moderate and severe hallux valgus and using weight‐bearing three‐dimensional assessments are required.

This study had some limitations. First, Thiel‐embalmed cadavers were used. Although Thiel fixation has been reported to preserve joint motion and some mechanical properties of muscles, tendons, and ligaments similar to those of living tissue [12, 23], it does not perfectly replicate in vivo conditions. Studies involving living participants are required to confirm these findings and assess their reproducibility. Second, because cadaveric specimens were used, HVA was evaluated under non‐weight‐bearing conditions. Although the standardized conditions permitted comparisons among specimens, the measurements are not directly equivalent to clinical weight‐bearing radiographic HVA. Third, this was a cross‐sectional observational study; therefore, causal relationships among PTML morphology, articular morphology, HVA, and TMT joint displacement could not be established. Fourth, the cadavers were of advanced age, which may limit the generalizability of the findings. Age‐related changes in ligament stiffness, collagen organization, articular cartilage, and bone morphology may have influenced both PTML morphology and TMT joint displacement. Therefore, the present findings may not be directly applicable to younger individuals or clinical populations with hallux valgus. Fifth, the sample size was small, particularly for Type III morphology, which included only five feet. Although linear mixed‐effects models were used to account for within‐cadaver clustering, the limited number of cadavers resulted in low statistical power and wide confidence intervals. Therefore, the absence of statistically significant findings should not be interpreted as evidence of no association. Sixth, stress was applied manually until resistance was perceived and was therefore not quantitatively standardized. Although high intra‐rater, inter‐session reliability was observed, inter‐examiner reliability was not evaluated, and examiner‐dependent variability may have influenced the measurements. Furthermore, the validity of this loading method against an established measure of first TMT joint instability was not evaluated. Seventh, ultrasonography quantified displacement in individual imaging planes and did not assess the three‐dimensional movement of the first TMT joint. Therefore, the measurements may not fully represent intrinsic three‐dimensional mobility or instability. Finally, the specimens showed a narrow HVA distribution: 17 of 18 feet had an HVA below 15°, and no specimen had moderate or severe hallux valgus. Consequently, the study could not meaningfully evaluate associations across the full range of hallux valgus severity, and the findings should not be generalized to patients with moderate or severe deformity.

## 5 Conclusion

In this small exploratory cadaveric study, which predominantly included feet without moderate or severe hallux valgus, no statistically significant associations were identified between first TMT joint displacement and articular surface morphology, PTML morphology, or HVA after accounting for within‐cadaver clustering and multiple comparisons. These findings should not be generalized to patients with moderate or severe hallux valgus. Larger studies using quantitatively standardized loading and three‐dimensional weight‐bearing assessments are required to clarify these relationships.

## Author Contributions

**Mutsuaki Edama:** conceptualization, methodology, investigation, writing – original draft preparation, and writing – review and editing. **Hiroki Tagawa:** conceptualization, methodology, investigation, writing – original draft preparation, and writing – review and editing. **Kaito Iwasaki:** formal analysis and writing – review and editing. **Kodai Sakamoto:** formal analysis and writing – review and editing. **Rinka Goto:** formal analysis and writing – review and editing. **Miyu Watanabe:** formal analysis and writing – review and editing. **Tomonobu Ishigaki:** writing – review and editing. **Makoto Komiya:** writing – review and editing. **Hirotake Yokota:** writing – review and editing. **Ryo Hirabayashi:** writing – review and editing. **Tomoya Takabayashi:** writing – review and editing. **Noboru Sato:** supervision, formal analysis, and writing – review and editing.

## Funding

This study was supported by Grants‐in‐Aid for Scientific Research (Grants 23H03258, 23KK0180, and 22K19739) from the Japanese Society for the Promotion of Science (JSPS).

## Ethics Statement

All experiments were performed in accordance with the 1964 Declaration of Helsinki, and all cadavers were donated for study purposes to the Niigata University Graduate School of Medical and Dental Sciences in Niigata, Japan. This study was approved by the Ethics Committee of the Niigata University Graduate School of Medical and Dental Sciences. Informed consent was obtained from the families of all participants.

## Consent

The authors have nothing to report

## Conflicts of Interest

The authors declare no conflicts of interest.

## Data Availability

The datasets generated and/or analyzed during the current study are not publicly available because of ethical restrictions concerning cadaveric donor data but are available from the corresponding author upon reasonable request.
